# Emotional stress recognition using a new fusion link between electroencephalogram and peripheral signals

**Published:** 2015-07-06

**Authors:** Seyyed Abed Hosseini, Mohammad Ali Khalilzadeh, Mohammad Bagher Naghibi-Sistani, Seyyed Mehran Homam

**Affiliations:** 1 Center of Excellence on Soft Computing and Intelligent Information Processing AND Department of Electrical Engineering, Ferdowsi University of Mashhad, Mashhad, Iran; 2 Research Center of Biomedical Engineering, Islamic Azad University, Mashhad Branch, Mashhad, Iran; 3 Department of Medical, Islamic Azad University, Mashhad Branch, Mashhad, Iran

**Keywords:** Electroencephalogram, Emotional Stress, Signal Processing, Recognition, Support Vector Machine

## Abstract

**Background:** This paper proposes a new emotional stress assessment system using multi-modal bio-signals. Electroencephalogram (EEG) is the reflection of brain activity and is widely used in clinical diagnosis and biomedical research.

**Methods: **We design an efficient acquisition protocol to acquire the EEG signals in five channels (FP1, FP2, T3, T4 and Pz) and peripheral signals such as blood volume pulse, skin conductance (SC) and respiration, under images induction (calm-neutral and negatively excited) for the participants. The visual stimuli images are selected from the subset International Affective Picture System database. The qualitative and quantitative evaluation of peripheral signals are used to select suitable segments of EEG signals for improving the accuracy of signal labeling according to emotional stress states. After pre-processing, wavelet coefficients, fractal dimension, and Lempel-Ziv complexity are used to extract the features of the EEG signals. The vast number of features leads to the problem of dimensionality, which is solved using the genetic algorithm as a feature selection method.

**Results: **The results show that the average classification accuracy is 89.6% for two categories of emotional stress states using the support vector machine (SVM).

**Conclusion:** This is a great improvement in results compared to other similar researches. We achieve a noticeable improvement of 11.3% in accuracy using SVM classifier, in compared to previous studies. Therefore, a new fusion between EEG and peripheral signals are more robust in comparison to the separate signals.

## Introduction

Emotions are complex phenomena that play a significant role in the quality of human life.^1^ Emotions are part of any natural communication among humans, generally considered as non-verbal cues.^2^ When thinking about emotional stress recognition systems, one of the applications that generally come to mind is the lie detector, but this is just the top of the iceberg and that many more applications can be targeted by research on emotional stress assessment.^3^ Emotional stress is psychology condition, which affects the central nervous system.^1^ In assessment of emotions, brain activity plays a central role. Emotion plays a major role in motivation, perception, cognition, creativity, attention, learning, and decision-making.^1^^,^^4^^,^^5^ Electroencephalogram (EEG) is the reflection of brain activity and is widely used in clinical diagnosis and biomedical researches. Researchers have found that the following frequency bands of EEG signals are interesting to be interpreted such as delta (1-4 Hz), theta (4-8 Hz), alpha (8-13 Hz), beta (13-30 Hz), and gamma (> 30 Hz).^1^^,^^6^ EEG signals inherently associated with perceptible characteristics that change in different situations will change. Thus, by extracting these features and analyzing them, it is possible to get a right perception about the nervous system.

A lot of research has been undertaken in the assessment of stress and emotion over the last years. The main reason is the fact that those feelings are present in many situations where humans are involved. Stress is often defined as the body’s reaction to a perceived mental, emotional or physical distress. Psychologists don’t agree on what is considered an emotion and how many types of emotions exist.^7^ Kleinginna gathered 92 definitions of emotion from literature present that day. He concluded that emotion is a complex set of interactions among subjective and objective factors, mediated by neural/hormonal systems.^8^ There are two main approaches to the definition of basic emotions: The biological view that is strongly anchored in the Darwinian and the Jamesian theories, and the psychological view.^9^ The most well-known theory represents emotions in two or three dimensional spaces, originating from cognitive theories, where valence-arousal space in emotions is expressed as a combination of two continuous variables: valence ranging from negative to positive (or unpleasant to pleasant) and arousal extending from calm to excited.^10^

In recent years, higher order spectra, wavelet coefficients, and chaotic invariants have received increasing interest in some of the applications.^1^^,^^11^^-^^14^ Most of researches in the domain of stress use peripheral signals such as respiratory rate, skin conductance (SC), blood volume pulse (BVP),^15^ and temperature.^16^ Previous studies have investigated the use of peripheral and brain signals separately, but little attention has been paid so far to the fusion between brain and peripheral signals.^1^^,^^3^^,^^11^^,^^17^

An important issue in every cognitive system is the correct labeling of the data. Here, labeling means the assessment of the data using a series of visual criteria used by psychologists and a proposed cognitive system for peripheral signals in order to verify the existence of a close correlation of the data and the psychological state of the subject. In this kind of research, putting the subject in the desired psychological state is very important. Most of the previous research performed in this field would expect the desired state only based on the assumption that the correct stimulus would bring it about. However, one needs to consider a lot of interfering parameters that can affect the mental and cognitive state of the subject individual, which will possibly result in not being in the desired state. As a result, many of the errors in emotional state recognition systems can be related to the lack of substantiating the existence of a close correlation of the data and the psychological state of the subject.

In this research, in addition to the stimulus, the output responses of the autonomic nervous system (ANS) or in other word the peripheral signals are used as the confirming characteristics to improve the labeling process. In other words, the desired psychological state of the subject is validated by qualitative and quantitative analysis of the peripheral signals.

This part provides a list of relevant studies concerning emotion assessment from bio-signals. In one study, Aftanas et al.^18^ showed significant differentiation of arousal based on EEG data collected from participants watching high, intermediate, and low arousal images. Chanel et al.^10^ asked the participants to remember past emotional events, and obtained the result of 79% using EEG signals and 53% using peripheral signals for three categories, 76% using EEG signals, and 73% using peripheral signals for two categories. In another study, Chanel^3^ asked the participants to remember past emotional episodes, and obtained the result of 88% using EEG for three categories with support vector machine (SVM) classifier. Furthermore, their results showed that, the importance of EEG signals for emotion assessment by classification as they had better accuracy than peripheral signals on the 8 s of recorded signal. Hosseini et al.^5^ used the induction visual images for recording the bio-signals in stimulate participants with two different emotions, resulting in 70% of correctly identified patterns, using EEG signals for two categories of emotional stress states. Their results showed that the EEG signals performed equally well as the peripheral signals, but a combination of both improved the results. In another study, Hosseini et al.^19^ used the induction visual images based acquisition protocol for recording the EEG and peripheral signals under two categories of emotional stress states of participants, and obtained the result of 78.3% using EEG signals with SVM classifier. Kim et al.^20^ used the combination of music and story as stimuli and there were 50 participants, to introduce a user independent system, the results were 78.4%, 61% for three and four categories of different emotions respectively. Takahashi^21^ used film clips to stimulate participants with five different emotions, resulting in 42% of correctly identified patterns. Schaaff et al.^22^ used pictures from the International Affective Picture System (IAPS) to induce three emotional states: pleasant, neutral, and unpleasant. They obtained the result of 66.7% for three classes of emotion, solely based on EEG signals.

The main goal of this research is to produce a new fusion link between peripheral and EEG signals for emotional stress states recognition in terms of quality and quantity. We investigated the recognition of two emotional stress states (calm-neutral and negatively excited) using SVM classifier.

The layout of the paper is as follows: Section 2 presents briefly the data acquisition protocol and labeling process of EEG signals. The methods and materials are given in Section 3. The results are covered in Section 4. The discussion is presented in Section 5. Finally, the conclusion is provided in Section 6.

***Acquisition protocol***

*Stimuli*

Every standard test in stress and emotion recognition has its own advantages and disadvantages.^1^ Most experiments that measure emotion from EEG signals use pictures from the IAPS.^23^ The IAPS evaluated by several American participants on two dimensions of nine points each (1-9). In this study, we chose the picture presentation test, based on the closeness of valence and arousal scores. The stimuli to elicit the target emotions (calm-neutral and negatively excited) are some of the pictures. The valence dimension ranging from negative to positive and the arousal dimension, ranging from calm to excited. The images in these classes are picked according to the rules in (1). Particular images, for example, erotic images duo to ethical considerations are removed from the selection.


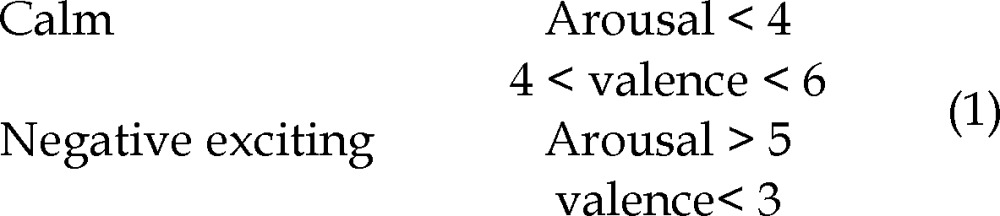


**Figure 1:**
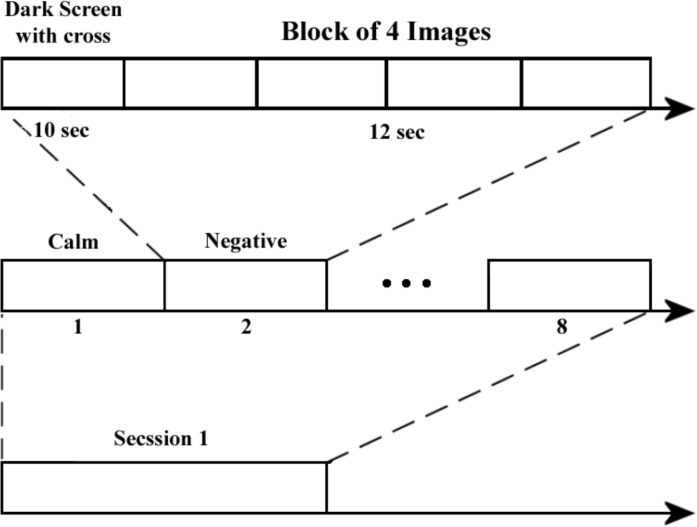
The protocol of data acquisition

The participant sits in front of a portable computer screen in a bare room relatively, the images to inform him about the specific emotional event he has to think of. Each experiment consists of 8 trials. Each stimulus consists of a block of four pictures, which ensures the stability of the emotion over time. In addition, each picture is displayed for 3 s leading to a total 12 s per block. Prior to displaying images, a dark screen with an asterisk in the middle is shown for 10 s to separate each trial and to attract the participant’s attention. The detail of each trial is shown in figure 1.

**Figure 2:**
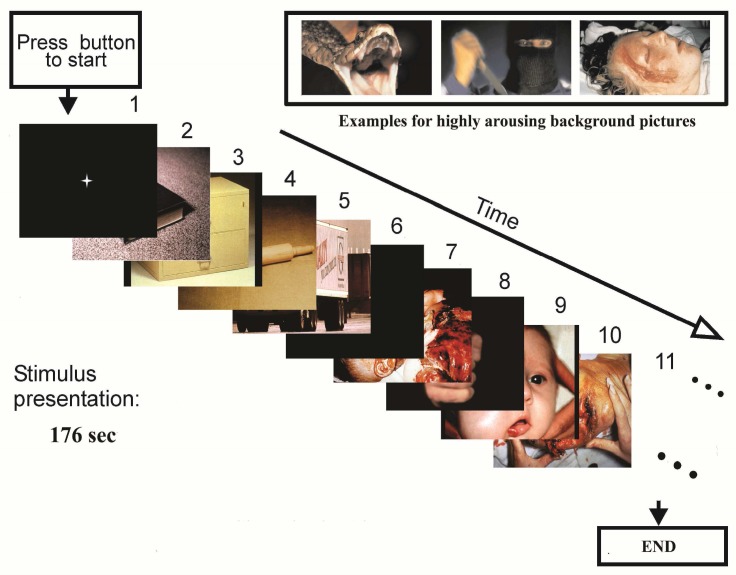
Process of picture presentation test

This epoch duration is chosen because to avoid participant fatigue. In figure 2, each presentation cycle started with a black fixation cross, which is shown for 10 s. After that pictures are presented for 12 s.

*Subjects*

Fifteen healthy volunteered subjects are right-handed males between the age of 20 and 24 years. Most subjects are students from Islamic Azad University in Mashhad Branch. Each participant is examined by a dichotic listening test to identify the dominant hemisphere.^1^^,^^24^ All subjects have normal or corrected vision; none of them have neurological disorders. These are performed to eliminate any differences in subjects. All participants gave written informed consent.^1^ Then each participant is given a particulars questionnaire.^1^ During the pre-test, several questionnaires have been evaluated in order to check the best psychological input to start the protocol phase; this test is state-trait anxiety inventory.^1^ At the end of the experiment, participants are asked to fill in a questionnaire about the experiment and give their opinions. Because, it is possible that the emotion that a participant experiences differs from the expected value. For that reason, the participant is asked to rate his emotion on a self-assessment.

We used a 10 channel Flexcom Infiniti device, with 14-bit resolution for data acquisition.^25^ It is connected to a PC using the USB port. An optical cable connects to device, to prevent any electrical charge from reaching the participant. The Flexcom Infiniti hardware only worked well with the accompanying software. Two programs are available, Biograph Infiniti Acquisition and ezscan. The central activity is monitored by recording EEGs. The peripheral activity is assessed using the following sensors: A SC sensor to measure sudation; a respiration belt to measure abdomen expansion; a plethysmograph to record BVP. We recorded SC by positioning two dedicated electrodes on the top of left index and middle fingers. The sample rate of the BVP and SC signals acquisition is 2048 Hz and respiration signal acquisition is 256 Hz. For reduce of calculation volume, are implemented the downsampling on BVP and SC signals. EEG is recorded using electrodes placed at five positions. The scalp EEG is obtained at location FP1, FP2, T3, T4, and Pz, as defined by the international 10-20 system. In order to measure a reference signal that is (as much as possible) free from brain activity, we have two electrodes to attach to the participants earlobes. The sample rate of the EEG signal acquisition is 256 Hz. Each recording lasted about 3 min. More details of the data acquisition protocol can be found in Hosseini.^1^

*Labeling process of EEG signals*

In order to choose the best emotional stress related to EEG signals, we implemented a new emotion-related signal recognition system, which has not been studied so far.^1^^,^^11^ We recorded peripheral signals concomitantly in order to firstly recognize the related to emotional stress state and then label the correlated EEG signals. In other words, we used the peripheral signals as a tutor for labeling system.

**Figure 3:**
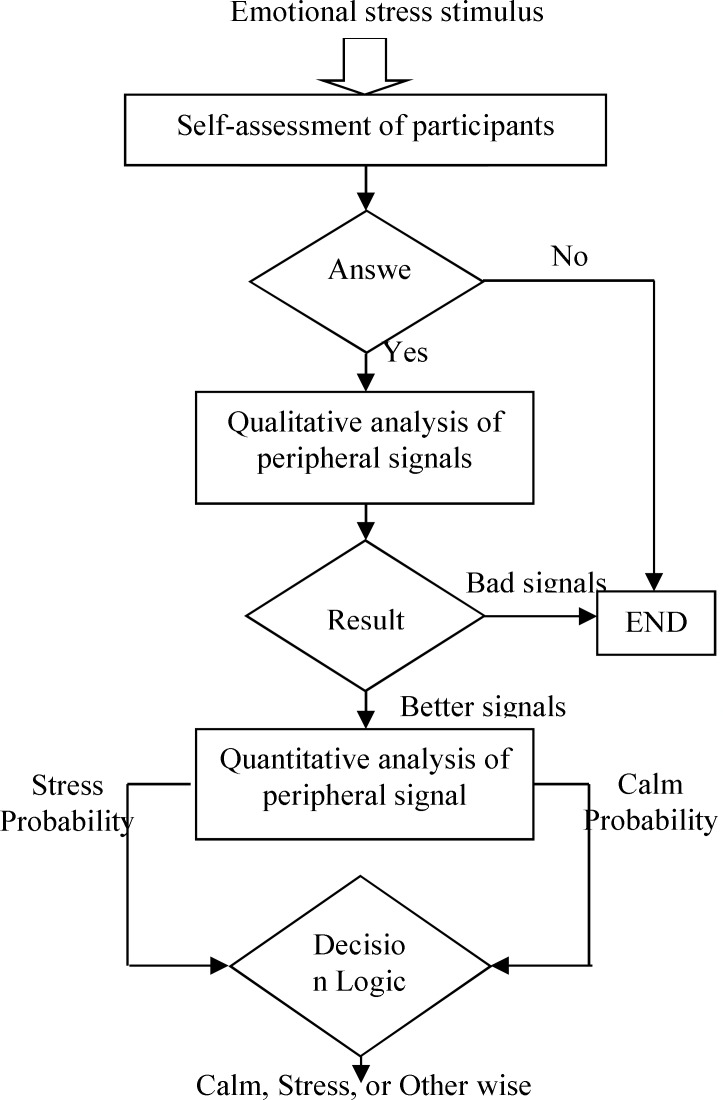
Labeling process of electroencephalogram signals

The process of labeling EEG signals consists of three stages: First self-assessment, second the qualitative analysis of peripheral signals, and third the quantitative analysis of peripheral signals. Figure 3 shows the different stages of the process. After the experiment, there is also a self-assessment stage, which is a good way to have an idea about the emotional stimulation “level” of the subject, because emotions are known to be very subjective and dependent on previous experience.^26^ In this research, we will be able to get a general idea of the quality of the data, i.e. if the data are good or bad.

One kind of this data is respiration. Emotional stress processes influence respiration.^27^^,^^28^ Slow respiration, for example, is linked to relaxation while irregular rhythm, quick variations, and cessation of respiration correspond to more aroused emotions like anger or fear.^10^ Another one is SC, which measures the conductivity of the skin. Since sweat gland activity is known to be controlled by the sympathetic nervous system, electrodermal activity has become a common source of information to measure the ANS. SC increases if the skin is sweaty, for example, when one is experimenting emotions such as stress. Moreover, blood pressure and heart rate variability (HRV) are variables that correlate with defensive reactions, pleasantness of a stimulus, and basic emotions.^10^ We obtained HR signal using BVP signal recorded by a plethysmograph. A method to determine HR from a BVP signal is proposed in Wan and Woo.^29^ Analysis of HRV provides an effective way to investigate the different activities of ANS, an increase of HR can be due to an increase of the sympathetic activity or a decrease of the parasympathetic activity. Two frequency bands (HR spectrum) are generally considered for HR signal, a low frequency band ranging from 0.05 Hz to 0.15 Hz and a high frequency band including frequencies between 0.15 Hz and 1 Hz.^1^ In order to analyze the peripheral signals quantitatively, we need to pre-process them, to remove environmental noises by applying filters. The peripheral signals are filtered by moving average filters to remove noise.

We used a common set of feature values for analysis of the peripheral signals (Table 1).^1^^,^^17^ The respiration features are from time and frequency domains, the SC features and the BVP features are from time domain, and the HRV features are from time, frequency domains, and fractal dimension.

After extracting the features, we need to classify them using a classifier. There are several approaches to apply the SVM for multiclass classification.^30^ The LibSVM toolbox is used for implementation of the SVM by one-versus-all method.^31^ Two SVMs that correspond to each of the two emotions are used. The i_th_ SVM is trained with all of the training data in the i_th_ class with calm labels, and the other training data with negative labels.

**Figure 4:**
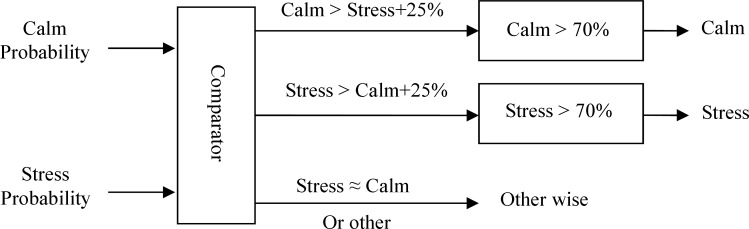
Decision logic algorithm to select the best emotional stress states

In the emotional stress recognition process, the feature vector is simultaneously fed into all SVMs and the output from each SVM is investigated in the decision logic algorithm to select the best emotional stress states (Figure 4). In the SVM classifier, is used a radial basis function (RBF) as a kernel function. RBF projects the data to a higher dimension.

A confusion matrix will also be used to determine how the samples are classified in the different classes. A confusion matrix gives the percentage of samples belonging to class ω_i_ and classified as class ω_j_. The accuracy can be retrieved from the confusion matrix by summing its diagonal elements P_i,j_ weighted by the prior probability P (ω_i_) of occurrence of the class ω_i_. The confusion matrices results of the SVM used for the classification of the peripheral signals under two emotional stress states is given in table 2.

The results show that, the classification accuracy with peripheral signals is 76.95% for the two categories, using SVM classifier with RBF kernel. The numbers of rejected trials that are badly classified that is lower than the number of correctly classified. The percentage of rejected trials is 11%. Method at this stage it has been used to select suitable segments of EEG signal for improving the accuracy of signal labeling according to emotional stress state. More details of the labeling process can be found in Hosseini.^1^

**Table 1:** Features extracted from peripheral signals

**Signal**	**Extracted features**
Respiration	Mean, variance, SD, Kurtosis, Skewness, maximum minus minimum value, power in the 0 to 2 Hz (∆f = 0.5 Hz) bands
SC	Mean, variance, SD, Kurtosis, Skewness, maximum, mean of derivative, energy response and proportion of negative samples in the derivative versus all samples
BVP	Mean, variance, SD, Kurtosis, Skewness, mean of trough variability, variance of trough variability, mean of peak variability, variance of peak variability, mean of amplitude variability, variance of amplitude variability, mean value variability, variance of mean value variability, mean of baseline variability, variance of baseline variability
HRV	Mean, variance, SD, low power frequency of 0.05-0.15 Hz, proportion low power frequency versus all power frequency, fractal dimension

SD: Standard deviation; SC: Skin conductance; BVP: Blood volume pulse; HRV: Heart rate variability

**Table 2:** The confusion matrices across participants using peripheral signals using radial basis function (RBF) kernel of support vector machine (SVM)

**Truth**	**Classified with SVM**	
	**Calm-neutral (%)**	**Negative excited (%)**
Calm-neutral	65.4	34.6
Negative excited	11.5	88.5

SVM: Support vector machine

## Materials and Methods

Before analysis, we first remove the data segment, which contains eye blinking, environmental noises, and drifts. The data are filtered using a band pass filter in the frequency band of 0.5~40 Hz.

Feature extraction is the process of extracting useful information from the signal. Features are extracted for each channel of EEG signals using wavelet coefficients, fractal dimension, and Lempel-Ziv complexity.

Petrosian fractal dimension (PFD) is generally used due to its quick estimation.^32^ In this method, a signal is produced by subtracting consecutive samples from the waveform record. From this sequence of subtractions, a binary sequence is created assigning +1 or −1 if the result of the subtraction is positive or negative, respectively. In short, PFD is defined as follows,


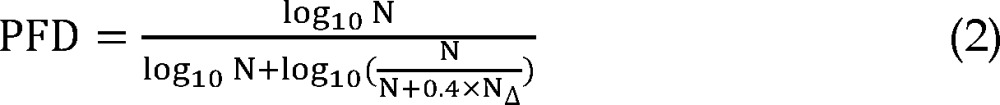


Where N and N_∆_ are the number of points of the sequence and the number of sign changes (number of dissimilar pairs) in the binary sequence generated, respectively. In this research, the best results are obtained for estimating the fractal dimension of the EEG; N = 512 samples (2 s) and window overlap = 0%.

Lempel and Ziv proposed a measure of the complexity of EEG recordings in 1976.^33^ Lempel-Ziv complexity counts the number of different patterns in a sequence, starting from short patterns to longer ones. In this study, Lempel-Ziv complexity is used for EEG analysis, since it can effectively characterize the development of spatiotemporal activity patterns in non-linear systems of high-dimensionality,^34^ such as the brain. Moreover, the concept of C(n) is simple to understand and its computation is easy. Before calculating Lempel-Ziv complexity, the signal must be transformed into a finite symbol sequence P. Here, a signal is transformed into a binary sequence (i.e. a 0-1 sequence) as follows,


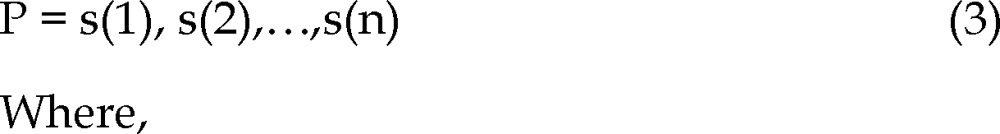



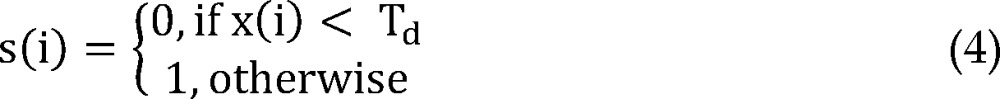


Usually, the median is used as the threshold T_d_ because of its robustness to outliers. A value which is below or equal to the mean of the data is represented by “0” and a value which is above the mean of the data is represented by “1.” The complexity of a random sequence with length n, b(n), for a sequence which consists of different binary codes with equal probability can be calculated as:


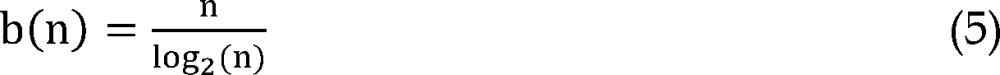


The normalized Lempel-Ziv complexity that reflects the arising rate of new patterns in the sequence, C(n), is obtained as:


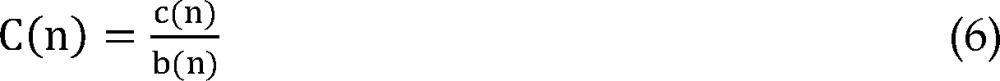


Discrete wavelet transform (DWT) based feature extraction has been successfully applied with promising results in physiological pattern recognition applications.^13^ Choice of suitable wavelet and the number of levels of decomposition is very important in the analysis of signals using DWT. In this study, we used Daubechies wavelet function with order db4 for extracting the statistical feature from the EEG signal. The number of levels of decomposition is chosen based on the dominant frequency components of the signal. The levels are chosen such that those parts of the signal that correlate well with the frequencies required for classification of the signal are retained in the wavelet coefficients. Since the EEG signals do not have any useful frequency components above 32Hz, the number of levels is chosen to be five. Thus, the signal is decomposed into the details D1-D5 and one final approximation, A5. The range of various frequency bands are shown in table 3.

**Table 3:** Frequencies corresponding to different levels of decomposition for “db4” wavelet with a sampling frequency of 256 Hz

**Decomposition ** **levels**	**Frequency ** **bandwidth (Hz)**	**Frequency bands**
D1	64-128	Noises
D2	32-64	Noises (gamma)
D3	16-32	Beta
D4	8-16	Alpha
D5	4-8	Theta
A5	0-4	Delta

The extracted wavelet coefficients provide a compact representation that shows the energy distribution of the EEG signal in time and frequency. Table 2 presents frequencies corresponding to different levels of decomposition for db4 wavelets with a sampling frequency of 256 Hz. It can be seen from table 2 that the components A5 are within the delta (0-4 Hz), D5 are within the theta (4-8 Hz), D4 are within the alpha (8-13 Hz), and D3 are within the beta (13-30 Hz). Lower level decompositions related to higher frequencies have negligible magnitudes in a normal EEG. In order to further diminish the dimensionality of the extracted feature vectors; statistics over the set of the wavelet coefficients is used.

- Mean of the absolute values of the wavelet coefficients in each sub-band
- Average power of the wavelet coefficients in each sub-band
- Standard deviation of the wavelet coefficients in each sub-band.

These features are extracted for each channel, so the total number of features by this method is: [3×4] = 12.

In order to normalize the features in the limits of [−11] we used (7).


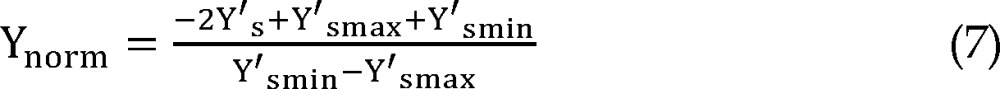


Here Y_norm_ is the relative amplitude.

**Figure 5:**
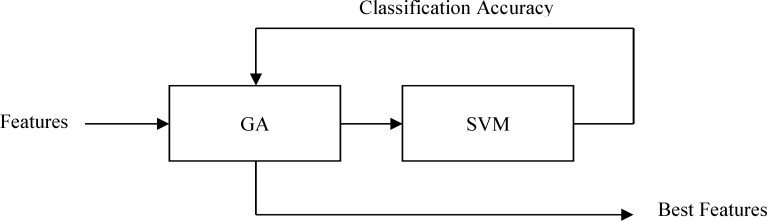
Combination of Genetic Algorithm and support vector machine to achieve the best features

GA: Genetic algorithm; SVM: Support vector machine

Genetic algorithm (GA) is one of the methods described for selecting appropriate features.^35^ The emphasis on using the GA for feature selection is to reduce the computational load on the training system while still allowing near optimal results to be found relatively quickly. The GA uses populations of 100 sizes, starting with randomly generated genomes. The probability of mutation is set to 0.01 and the probability of crossover is set to 0.4. The classification performance of the trained network using the whole dataset is returned to the GA as the value of the fitness function (Figure 5). We attempted to detect the feature sets related to negative/calm emotion response from EEG signal.

We used GA in assessment of all the features because a perfect feature group is not necessarily achievable by simply putting a few superior features since the data characteristics and features may have overlapping.

After extracting the desired features, we still have to find the related emotional stress states in the EEG. A classifier will do this process. SVM are maximum margin classifiers that try to maximize the distance between the decision surface and the nearest point to this surface. Non-linear SVM, maps the input space to a high dimensional feature space, and then constructs a linear optimal hyperplane in the feature space, which relates to a non-linear hyper-plane in the input space. The major problem of training machine is to find a kernel function that can not only capture the essential properties of the data distribution, but also prevent the over-fitting problem. We used three kernel functions including linear, polynomial, and RBF. The C parameter that regulates the tradeoff between training error minimization and margin maximization is empirically set to1 in this study.

## Results

In this research, we used a 2 s time intervals rectangular window without overlap, corresponding to blocks of 512 samples of EEG signals for data segmentation. In classification is important that the training set contain enough instances. On the other hand, it also important that the test set contains enough samples to avoid a noisy estimate of the model performance. We used around 75% of the EEG signals for the training, and 15% of the data for testing whether the learned relationship between the data and emotional stress is correct and the last 10% is used for validating the data. The results show that, the average classification accuracy with EEG signals is 89.6% for the two categories using the SVM classifier with RBF kernel. This is particularly true in our case since the number of emotional stimulations is limited by the duration of the protocols, which should not be too long to avoid participant fatigue, as well as elicitation of undesired emotions. 

## Discussion

Each standard test in stress assessment has its own advantages and disadvantages. We chose the picture presentation test, based on its valence and arousal scores in different psychological states. We have chosen the brain signals over the pure peripheral signals since that brain signals represent behavior directly from their source, but the peripheral signals are secondary manifestations of the ANS in response to emotional stress. Comparing the results of peripheral signals analysis, we notice that the breathing and SC signals are less reliable in accuracy compared to BVP and HRV signals. The results show that the classification accuracy with peripheral signals is 76.95% for two categories by SVM classifier with RBF kernel.

The process of labeling EEG signals consists of three stages: first self-assessment, second the qualitative analysis of peripheral signals and third the quantitative analysis of peripheral signals. After the experiment, there is also a self-assessment stage, which is a good way to have an idea about the emotional stimulation “level” of the subject, because emotions are known to be very subjective and dependent on previous experience. The only use of the personal moods and the subject’s self-assessment to confirm the quality of the registered brain signals can cause many errors. As a result, we need to use peripheral signals as a secondary trainer. In order to choose the best emotional stress state related to EEG signals, we implemented a new emotion-related signal recognition system, which has not been studied so far.^1^^,^^11^ Furthermore, we recorded peripheral signals continuously in order to first recognize the related to emotional stress state and then preferred label to EEG signal. Recent researches on the EEG signals, revealed the chaotic nature of this signal.^1^^,^^14^ It is logical not to use conventional methods that assume emotion can be analyzed by linear models. Because brain signals essentially have a chaotic non-linear behavior. We performed emotional stress assessment using both linear and non-linear features. Wavelet coefficients and chaotic invariants like fractal dimension and Lempel-Ziv complexity are used to extract the characteristics of the EEG signals. For most non-linear measures, a dimension should be defined to visualize the attractor in phase space. However, a problem associated with all of them is that defined dimension for the phase space is not constant either for all channels of recorded EEG signals or for different subjects. Depending on the conditions, the chosen dimension can be different. On the other hand, the performance of each measure can be depending on the values of dimension. Hence, using some equations and trial and error the optimum dimension for getting the best results can be discovered.

The results obtained of the fractal method indicate that a similar trend of reduction in fractal dimension value for the negative state compared to the calm state. The reduction in fractal dimension values characterizes the reduction in brain system complexity for participants with negative emotional stress state. Therefore, the number of the necessary dynamic equations for the description of the brain state in the negative emotional stress state experienced a decrease. A new approach to emotional stress states analysis using Lempel-Ziv complexity is described in this research. The results of analysis of the non-linear characteristics show that, if the parameters and the length of data are determined appropriately, the results can be a good representation of the brain behavior in emotional stress states.^1^ Hence, the application of non-linear time series analysis to EEG signals offers insight into the dynamical nature and variability of the brain signals. Therefore, it seems that non-linear features would lead to better understanding of how emotional activities work.

In this research, two of the advantages confirm the credibility of our results. We use dichotic hearing test and peripheral signals to label the brain signals correctly. Therefore, we can deduce that in short term data acquisition there is no specific dynamicity, which can be attributed to the short time intervals of 2 s. It is possible that by performing longer tests and using bigger intervals there is hope to identify some dynamics.

The results show that, the analysis of EEG signals for emotional stress assessment is better than peripheral signals.^10^ We used 2 s time intervals with rectangular window without overlap to analyze the brain signals, which resulted in a time resolution of 2 s in emotional stress states recognition. If we had used shorter time intervals with overlap, we could have achieved a greater but virtual time resolution. For example, it can be useful in biofeedback applications. The problem of high dimensionality is solved using GA as a feature selection method. The results show that the average classification accuracy is 89.6% for two categories of emotional stress states using the SVM classifier. In addition, it is shown that the new fusion link, between EEG and peripheral signals are more robust in comparison to the separate signals.^1^^,^^11^^,^^19^ This is a great improvement in results compared to other similar previous researches. Using proposed hybrid approach, we achieved a noticeable improvement of 11.3% in accuracy in comparison to previous studies.^19^

As a side result, many of the errors in emotional state recognition systems can be related to the lack of substantiating the existence of a close correlation of the data and the psychological state of the subject. Analyzing and comparing the results of previous researches is a complicated task, because the number of participants, the type of data, the method which is used and the time interval for analysis are different. Due to these differences, we cannot exactly compare our results with previous studies.

## Conclusion

In this research, we proposed a new approach to classify emotional stress in two main areas of the valance-arousal space using multi-modal bio-signals. EEG signals are the reflection of brain activity and are widely used in clinical diagnosis and biomedical research. These signals are used as a main signal. The visual stimuli images are selected from the subset IAPS database. The qualitative and quantitative evaluation of peripheral signals are used to select suitable segments of EEG signals for improving the accuracy of signal labeling according to emotional stress states. This is a great improvement in results compared to other similar researches. We achieve a noticeable improvement of 11.3% in accuracy using SVM classifier, in compared to previous studies. Therefore, a new fusion between EEG and peripheral signals are more robust in comparison to the separate signals.

## Notes:

**How to cite this article: **Hosseini SA, Khalilzadeh MA, Naghibi-Sistani MB, Homam SM Emotional stress recognition using a new fusion link between electroencephalogram and peripheral signals. Iran J Neurol 2015; 14(3): 142-51.
